# Supplementary material for: Elevated Levels of Growth/Differentiation Factor-15 in the Aqueous Humor and Serum of Glaucoma Patients
Source: J Clin Med. 2022 Jan 29;11(3):744. doi: 10.3390/jcm11030744 (PMC8837086; doi:10.3390/jcm11030744)
Supplement: Supplementary file 1 [file jcm-11-00744-s001.zip › jcm-1569755-supplementary.pdf]

Figure S1: Elevated IOP in POAG patients compared to non-glaucoma (cataract patients). The box and whisker plots represent median and the interquartile range in the distribution.

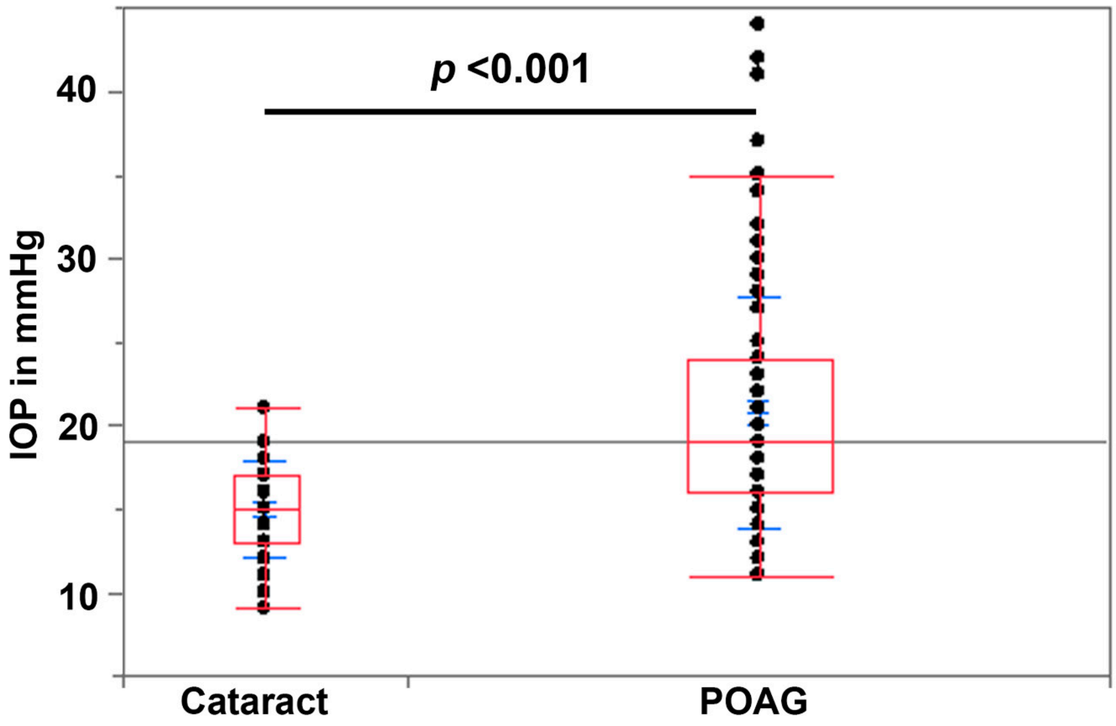

P-values were based on Wilcoxon rank sum test of difference between medians.

Figure. S2. Relationship between aqueous humor and serum GDF15 levels and IOP in POAG patients. AH GDF15 (**A**), serum GDF15 (**B**) and IOP of POAG patients did not reveal a significant relationship.

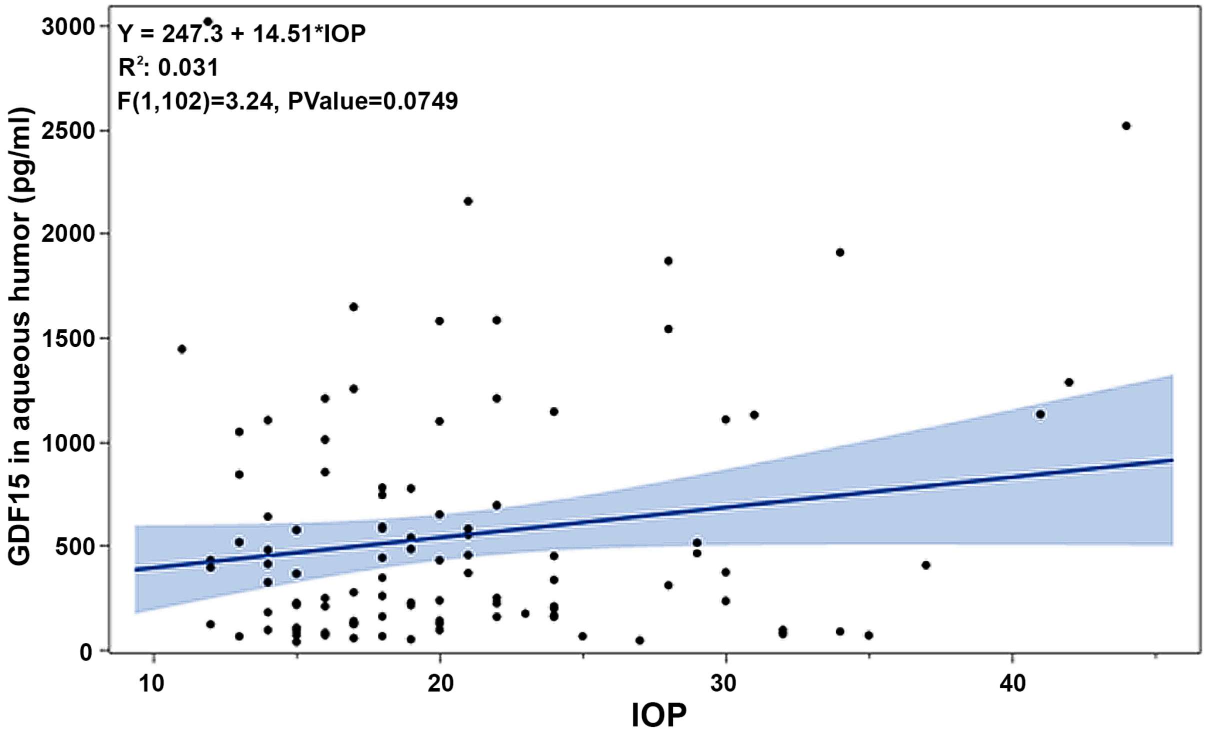

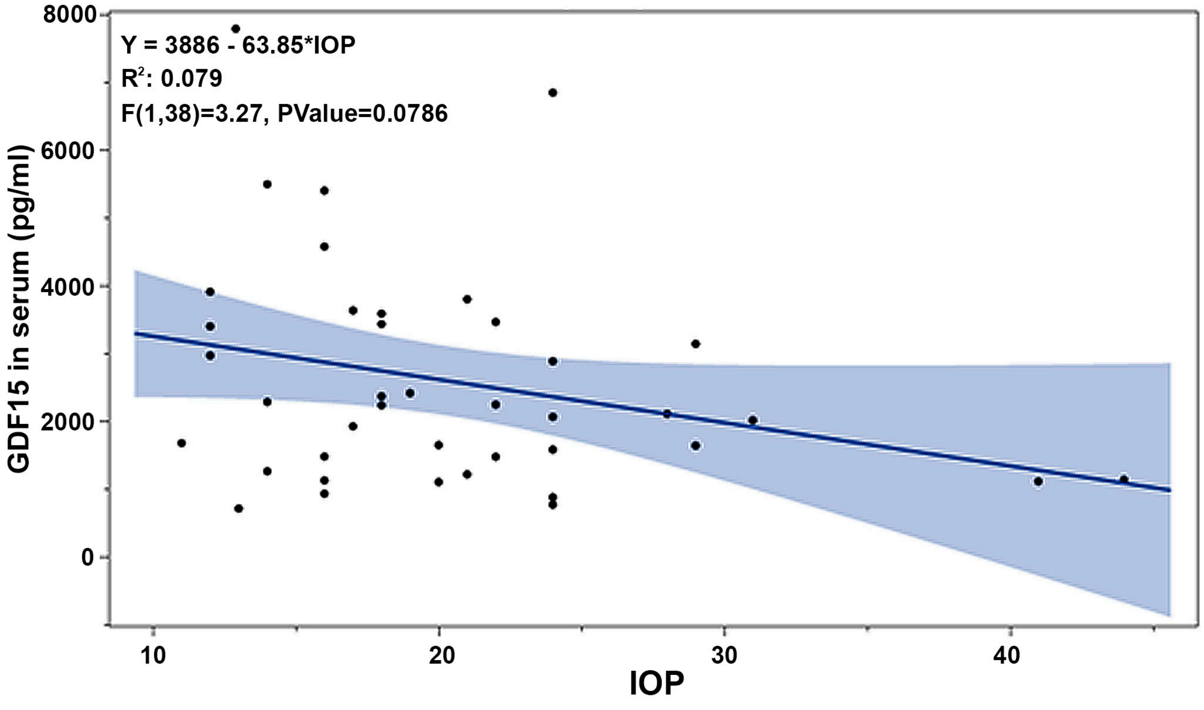

Figure S3. Relationship between aqueous humor and serum GDF15 levels and age in POAG patients. AH GDF15 (**A**), serum GDF15 (**B**) and age of POAG patients did not reveal a significant relationship.

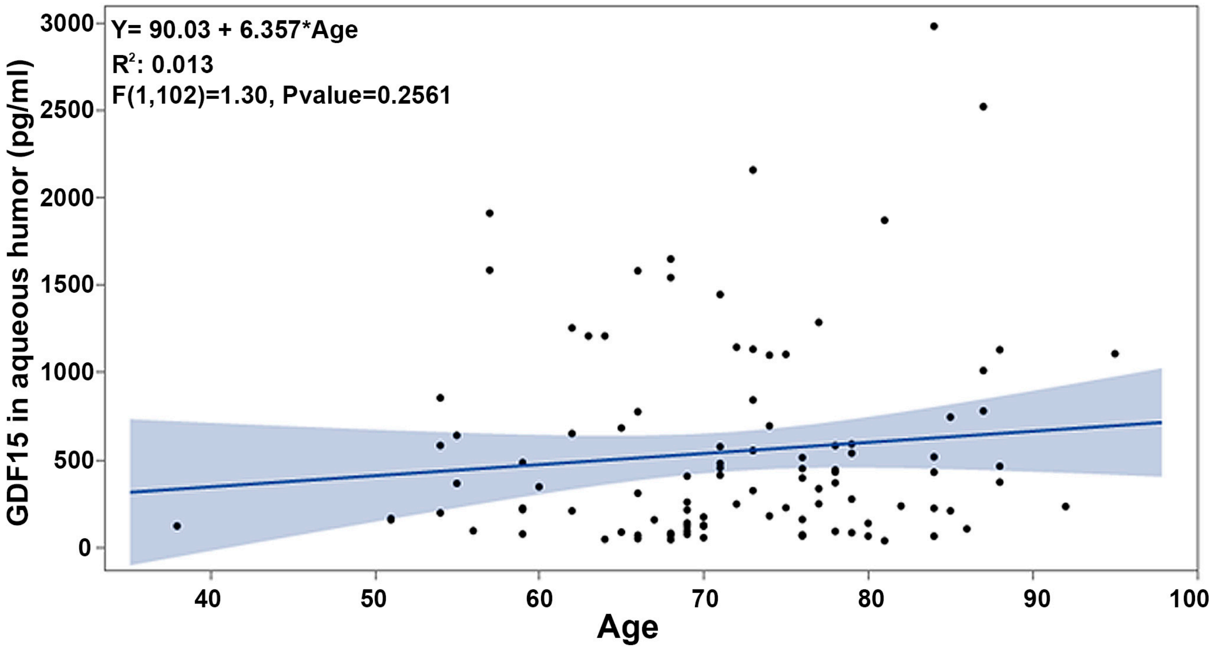

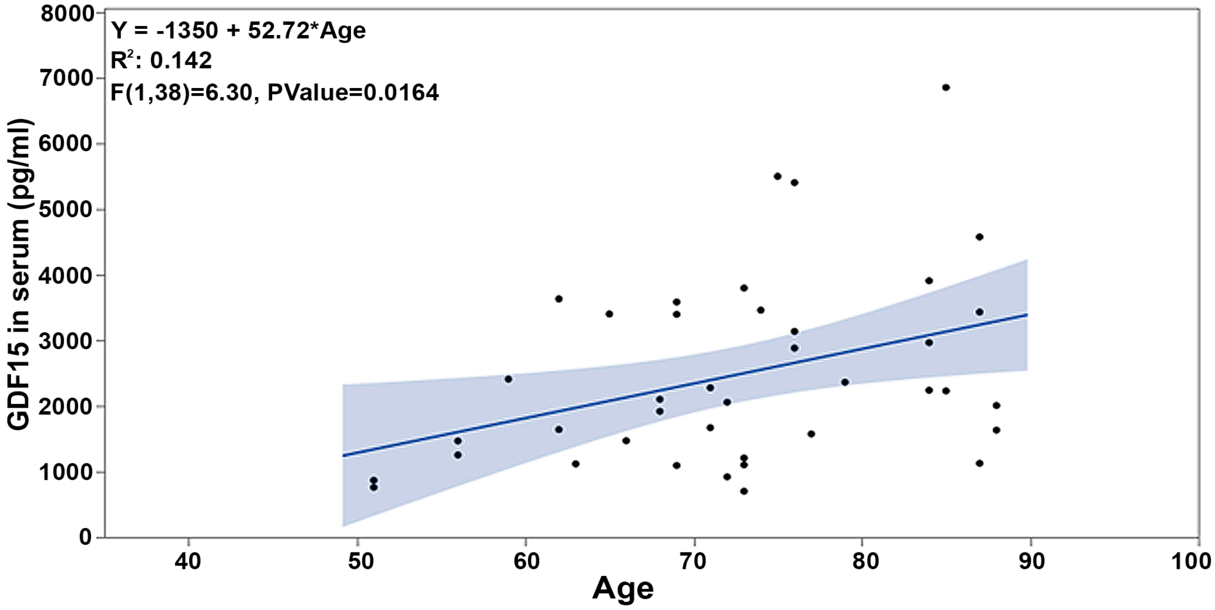

Table S1: Demography details of the subgroup of POAG and cataract patient study

		**Cataract n=32**	**POAG n=41**	***P*-value***
Age	n	32	40	
	Mean (SD)	70.8 (7.3)	72.6 (10.2)	0.416
	Min, Median, Max	51, 72, 84	51, 73, 88	
Gender	n (%)			
Female		13 (40)	22 (53)	
Male		19 (59)	19 (46)	0.346
Race	n (%)			
Asian		1 (3)	1 (2)	
Caucasian		29 (91)	19 (46)	
African American		2 (6)	21 (51)	<0.001

**P-value for gender and race based on Fisher's exact test.*

P-value for age based on two sample t-test.

Abbreviations: POAG, primary open-angle glaucoma; SD, Standard deviation.

Table S2: GDF15 levels in the aqueous humor and serum of POAG and non-glaucoma (cataract) subjects.

		Cataract	POAG	*P*-value*
GDF15 (AH)	n	32	40	
	Mean (SD)	103.63 (141.06)	791.20 (675.16)	
	Min, Median, Max	1.5, 63.5, 776.0	68.0, 619.0, 2978.0	<0.001
GDF15 (Serum)	n	32	41	
	Mean (SD)	1659.44 (1029.49)	2606.10 (1634.56)	
	Min, Median, Max	189.0, 1481.5, 3950.0	701.0, 2228.0, 7771.0	<0.011

**P*-value based on Wilcoxon rank sum test.
Abbreviations: GDF15, Growth/Differentiation Factor-15; POAG, primary open-angle glaucoma; AH, Aqueous humor; SD, Standard deviation.

Table S3: GDF15 levels in the aqueous humor and serum of male and female POAG and cataract patients.

Variable	Gender	Statistic	Cataract	POAG	*p*-value*
GDF15 (AH)	Female	n	19	21	
		Mean (SD)	89.37 (68.73)	750.19 (844.09)	
		Min, Median, Max	3.0, 76.0, 250.0	68.0, 411.0, 2978.0	<0.001
GDF15 (AH)	Male	n	24	19	
		Mean (SD)	104.88 (154.19)	836.53 (438.03)	
		Min, Median, Max	1.5, 64.0, 776.0	138.0, 743.0, 1647.0	<0.001
GDF15 (Serum)	Female	n	13	22	
		Mean (SD)	1241.62 (930.85)	2598.73 (1735.00)	
		Min, Median, Max	189.0, 1302.0, 3682.0	701.0, 2141.5, 6849.0	0.015
GDF15 (Serum)	Male	n	19	19	
		Mean (SD)	1945.32 (1017.50)	2614.63 (1557.31)	
		Min, Median, Max	287.0, 1850.0, 3950.0	1093.0, 2228.0, 7771.0	0.148

**P-value based on Wilcoxon rank sum test of difference between medians.*

Abbreviations: GDF15, Growth/Differentiation Factor-15; POAG, primary open-angle glaucoma; AH, Aqueous humor; SD, Standard deviation.

Table S4: Demography details of the large cohort POAG and cataract patient study

		Cataract N=117	POAG N=106	*P*-value*
Age	n	117	105	
	Mean (SD)	70.6 (8.7)	71.5 (10.3)	0.480
	Min, Median, Max	45, 72, 90	38, 71, 95	
Gender	n (%)			
Female		64 (55)	54 (51)	
Male		53 (45)	52 (49)	0.593
Race	n (%)			
Asian		4 (3)	1 (1)	
Caucasian		95 (82)	51 (49)	
African American		16 (14)	53 (50)	
Indian		1 (1)	0	<0.001

**p-value for gender and race based on Fisher's exact test.*

p-value for age based on two sample t-test.

Abbreviations: POAG, primary open-angle glaucoma; SD, Standard deviation.

Table S5: GDF15 levels in the aqueous humor of POAG and cataract patients.

		Cataract	POAG	*P*-Value*
GDF15 (AH)	n	117	105	
	Mean (SD)	69.32 (87.63)	550.18 (577.13)	
	Min, Median, Max	0.3, 49.0, 776.0	36.1, 364.0, 2978.0	<0.001

**p-Value based on Wilcoxon rank sum test of difference between medians.
Abbreviations: GDF15, Growth/Differentiation Factor-15; POAG, primary open-angle glaucoma; AH, Aqueous humor; SD, Standard deviation.

Table S6: GDF15 levels in aqueous humor and serum samples from male and female cataract patients.

Variable	Statistic	Female	Male	***P*-value***
GDF15 (AH)	n	64	53	
	Mean (SD)	60.41 (65.33)	80.07 (108.36)	
	Min, Median, Max	0.3, 39.0, 357.1	1.5, 52.0, 776.0	0.046
GDF15 (Serum)	n	13	19	
	Mean (SD)	1241.62 (930.85)	1945.32 (1017.50)	
	Min, Median, Max	189.0, 1302.0, 3682.0	287.0, 1850.0, 3950.0	0.025

**p-value based on Wilcoxon rank sum test of difference between medians.*

Abbreviations: GDF15, Growth/Differentiation Factor-15; AH, Aqueous humor; SD, Standard deviation.
